# Probing dark exciton diffusion using photovoltage

**DOI:** 10.1038/ncomms14215

**Published:** 2017-01-27

**Authors:** Tyler K. Mullenbach, Ian J. Curtin, Tao Zhang, Russell J. Holmes

**Affiliations:** 1 Department of Chemical Engineering and Materials Science, University of Minnesota, Minneapolis, Minnesota 55455, USA

## Abstract

The migration of weakly and non-luminescent (dark) excitons remains an understudied subset of exciton dynamics in molecular thin films. Inaccessible via photoluminescence, these states are often probed using photocurrent methods that require efficient charge collection. Here we probe exciton harvesting in both luminescent and dark materials using a photovoltage-based technique. Transient photovoltage permits a real-time measurement of the number of charges in an organic photovoltaic cell, while avoiding non-geminate recombination losses. The extracted exciton diffusion lengths are found to be similar to those determined using photocurrent. For the luminescent material boron subphthalocyanine chloride, the photovoltage determined diffusion length is less than that extracted from photoluminescence. This indicates that while photovoltage circumvents non-geminate losses, geminate recombination at the donor–acceptor interface remains the primary recombination pathway. Photovoltage thus offers a general approach for extracting a device-relevant diffusion length, while also providing insight in to the dominant carrier recombination pathways.

## Abstract (web summary)

Exciton diffusion plays a role in many optoelectronic devices. In some materials, this migration cannot be detected using photoluminescence. Mullenbach *et al*. use photovoltage measurements to extract the diffusion length in organic photovoltaic cells, and examine a series of non-luminescent materials.

As a quasi-particle with no net charge, excitons are difficult to probe directly by electrical means. This is particularly true in organic semiconductors where the exciton binding energy is ∼1 electronvolt^1^,^2^. As such, devices constructed using these materials are often intrinsically excitonic, and the properties of the exciton strongly determine device design and functionality^3^,^4^,^5^,^6^,^7^,^8^,^9^. Consequently, knowledge of exciton behaviour is essential in order to properly interpret material and device physics. This has been especially true in the evolution of organic photovoltaic cell (OPV) design. In an OPV, excitons must diffuse from the point of photogeneration to an electron donor–acceptor (D–A) interface in order to be dissociated and collected as a photocurrent^1^,^2^,^10^. Indeed, much effort has been directed at characterizing exciton transport and measuring the characteristic exciton diffusion length (*L*_D_). Such measurements often rely on tracking the end-of-life products of the exciton, namely, photons or charge carriers^11^,^12^,^13^,^14^,^15^,^16^,^17^,^18^,^19^.

In steady-state photoluminescence (PL)-based measurements of *L*_D_, emitted photons represent excitons that fail to reach the dissociating interface. The ratio of steady-state PL from a film with a dissociating interface to the PL from a film without a dissociating interface is equivalent to the ratio of exciton densities in the two films, and can be modelled using a diffusion equation to extract *L*_D_^3^,^11^,^12^,^20^,^21^,^22^. Measurements of transient PL can also reveal similar insights into exciton transport, based on a reduction in the exciton lifetime due to interaction with a quencher^16^,^23^,^24^. Despite the popularity of PL-based measurements of exciton transport, they are incapable of probing the dynamics of singlet or triplet excitons with exceedingly low luminescence quantum yields.

Charge carrier based techniques are capable of probing materials with low luminescence efficiencies, however these measurements can require involved experimental techniques, non-device relevant architectures or are subject to charge collection losses^2^,^18^,^19^,^20^,^25^. The most straightforward of these approaches are photocurrent-based measurements, where device external quantum efficiency (*η*_EQE_) spectra are fit for *L*_D_ by combining rigorous simulations of the generating optical field with a diffusion equation^2^,^20^,^25^. While excellent fits to the experimental data can be realized, losses due to geminate recombination at the D–A interface as well as non-geminate recombination during the charge collection process are often ignored^2^,^20^,^26^. This approximation can result in underestimates in the extracted value of *L*_D_ whenever the overall charge collection efficiency (*η*_CC_) <1. Fitting of *η*_EQE_ spectra is thus only expected to yield a value of *L*_*D*_ comparable to PL measurements in the absence of all recombination losses.

Instead of extracting carriers as photocurrent, we measure the number of carriers immediately after generation using photovoltage. Exciton dissociation leads to the initial formation of a charge transfer state at the D–A interface, followed by dissociation of the charge transfer state into mobile carriers. Indeed, dissociation of the charge transfer state may compete with rapid geminate recombination at the interface. Once dissociated, charge carriers occupy sites of disparate potential energy, leading to the generation of a measurable voltage. The magnitude of the voltage generated by an OPV is proportional to the number of charge carriers in the donor and acceptor active layers^27^,^28^,^29^. To measure *L*_D_ using a photovoltage-based technique, the exact relationship between the number of charge carriers and voltage must be known, and it must also be demonstrated that non-geminate recombination losses are negligible. Since charge transfer states that undergo rapid recombination will not accumulate in numbers sufficient to generate a measureable photovoltage, losses related to geminate recombination are included in the photovoltage measurement. Here the *L*_D_ of boron subphthalocyanine chloride (SubPc, Fig. 1a) and C_60_ are measured using photovoltage, and the value for SubPc is compared with previous PL-based measurements. The technique is then employed to measure *L*_D_ for several dark phthalocyanine materials, which are inaccessible using PL methods.

## Results

### Probing exciton diffusion with photovoltage

**Figure 1:**
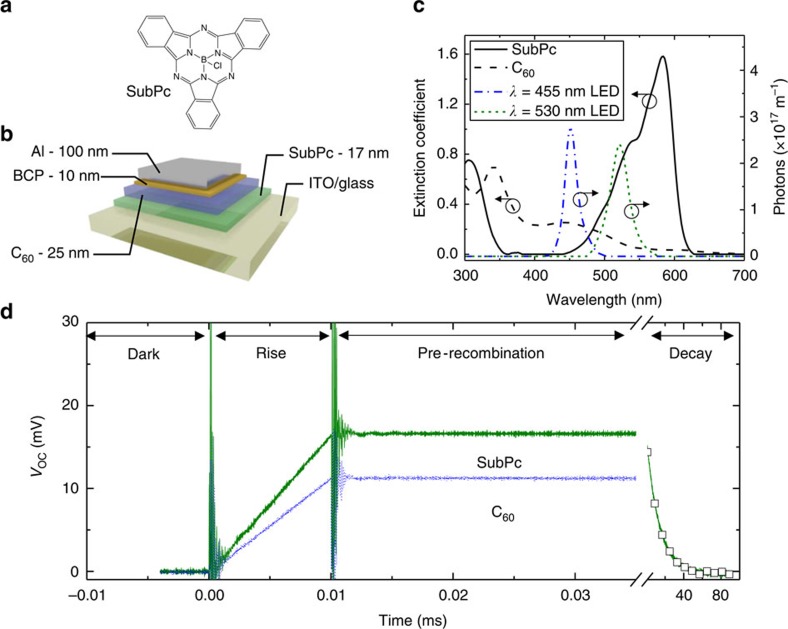
Photovoltage response for a SubPc-C_60_ planar heterojunction OPV. (**a**) Molecular structure of SubPc and (**b**) architecture for the SubPc-C_60_ OPV. (**c**) Pump spectra for LEDs peaked at wavelengths of *λ*=455 nm and *λ*=530 nm compared with the thin film extinction coefficients of SubPc and C_60_. (**d**) Representative plot of the open-circuit voltage response to a 10 μs incident light pulse showing four data sets. There are two data sets shown in the regions labelled Dark, Rise and Pre-recombination, where SubPc data is shown in green and C_60_ data is shown in blue. The region labelled ‘Decay' shows two photovoltage decays, one is for primarily pumping C_60_ (squares) and one for primarily pumping SubPc (green line in decay). Both decays correspond to a peak voltage of 16.5 mV and have time constants of ∼14 ms. The equivalence of the decays demonstrates that the photovoltage decay only depends on the number of carriers and not which material is being pumped.

Figure 1b shows the device architecture used to demonstrate the photovoltage-based technique for measuring *L*_D_. The OPV is a planar heterojunction device comprised of the electron donating material SubPc paired with the electron accepting material C_60_. The device is capped with an exciton blocking layer of bathocuproine (BCP) and a 100-nm-thick aluminum (Al) cathode. While the focus of this work lies in applying the photovoltage-based technique to dark materials, SubPc serves as a useful starting point since *L*_D_ has been previously measured by PL-based techniques^3^,^12^. For the device of Fig. 1b, SubPc or C_60_ can be probed by varying the pump light-emitting diode (LED) spectrum. The extinction coefficients of SubPc and C_60_ are shown in Fig. 1c, along with the emission spectra of two LEDs: a blue LED (*λ*_peak_=455 nm) used to primarily excite C_60_ and a green LED (*λ*_peak_=530 nm) used to excite SubPc. When the SubPc-C_60_ device is held at open-circuit and excited by one of the LEDs for 10 μs, the resulting photovoltage response is shown in Fig. 1d. Since SubPc and C_60_ do not have completely distinct extinction coefficients, excitons originating on SubPc comprise about 79% and 9% of the photovoltage response for the green and blue LED pulses, respectively^20^. The photovoltage response of Fig. 1d is characterized by four regions termed dark, rise, pre-recombination, and decay. In the dark region, the LED is off and there are no carriers present in the device, corresponding to zero photovoltage. During the rise phase, the LED is on, leading to exciton generation in the OPV, some of which undergo charge transfer at the D–A interface to generate charge carriers. The accumulation of charge carriers generates the linear voltage rise. Once generated, there is a rate for charge carriers to undergo recombination, leading to the photovoltage decrease shown in the decay region. The eventual decay in photovoltage is attributed to non-geminate charge carrier recombination, and is slow enough that a measure of the photovoltage can be made before any substantial number of charge carriers recombine. This is demonstrated by the photovoltage ‘plateau' observed at short times in the pre-recombination region of Fig. 1d.

The behaviour in Fig. 1d can be approximated by different regimes of the rate equation governing the number of carriers (*n*) in the OPV:


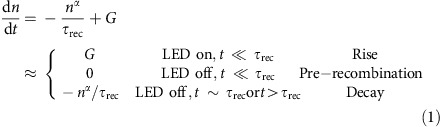


In equation (1), *G* is the generation rate of charge carriers, *τ*_rec_ is the non-geminate, charge carrier recombination time, and *α* represents the order of recombination^30^. The terms on the right of equation (1), refer to the corresponding regions of Fig. 1d. The plateau observed in Fig. 1d verifies that *τ*_rec_ is sufficiently long such that no significant charge carrier recombination occurs during the LED pulse. In other words, the plateau persists for a time longer than the LED pulse time. As long as the voltage is measured before the plateau begins to decay, only negligible recombination has occurred which enables an accurate measure of the number of charge carriers generated at the interface.

### Relating photovoltage to the number of charge carriers

In order to extract a diffusion length from photovoltage data, the curves of Fig. 1d must be translated into a measurement of the number of charge carriers present in the device. The relationship between photovoltage and the number of charge carriers is non-trivial to calculate^27^,^28^, but it can be directly measured using charge extraction methods^31^,^32^,^33^. While the charge extraction approach is employed here, any means of relating the number of charge carriers to the device voltage could be used instead of charge extraction. In a charge extraction experiment, the OPV is illuminated and held at open-circuit. Once the steady-state voltage is reached and recorded, the OPV is switched to short-circuit and the resulting current transient is recorded. The integral of the current transient yields the number of charge carriers extracted from the cell as well as the relationship between the number of carriers and the photovoltage^29^,^34^.

**Figure 2:**
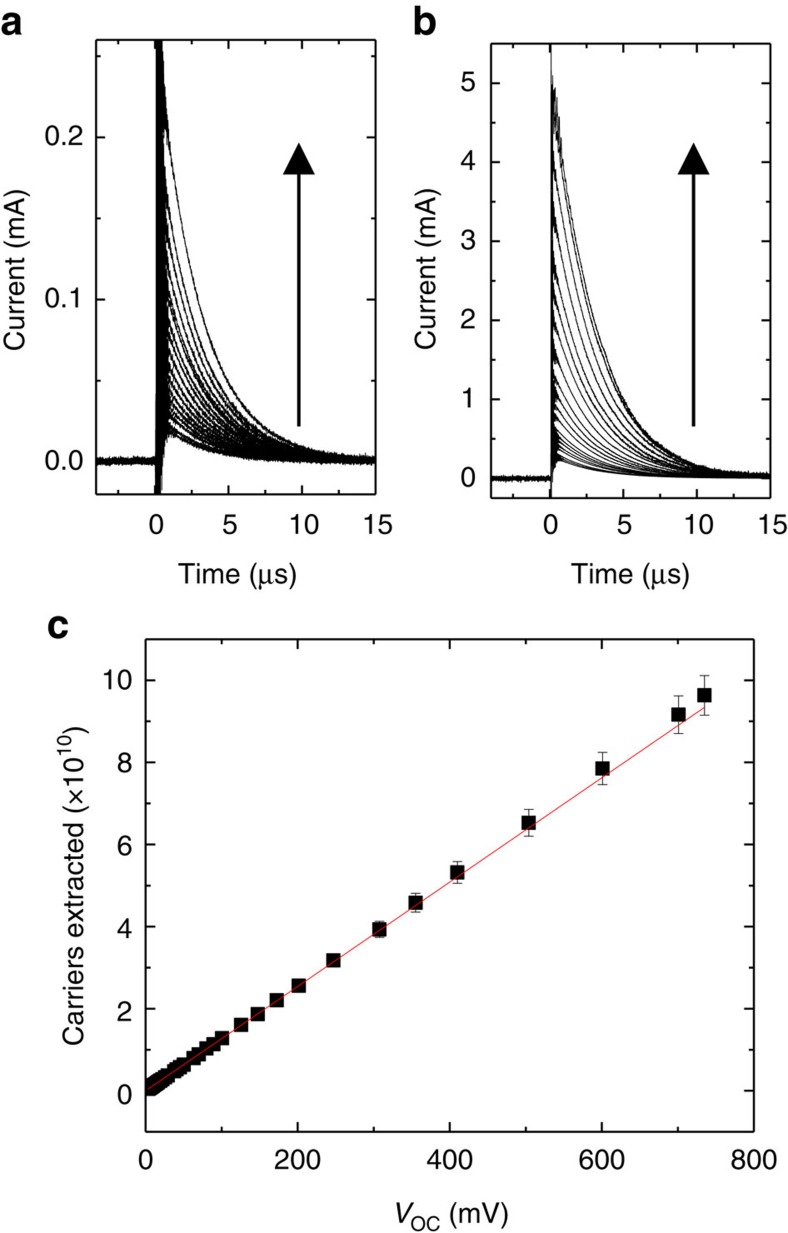
Charge extraction to relate photovoltage and number of charge carriers. (**a**,**b**) Current transients obtained by switching the SubPc-C_60_ device in Fig. 1b from open-circuit (steady-state) to short-circuit. The arrow points in the direction of increasing optical illumination and hence, *V*_OC_ before switching to short-circuit. In (**a**), the *V*_OC_ is varied from 3.4 mV to 37.6 mV while in (**b**), the *V*_OC_ is varied from 40.8 mV to 735 mV. (**c**) The trend of carrier number as a function of photovoltage obtained by integrating parts (**a**) and (**b**) with respect to time. A linear fit (solid red line) to the data is also shown with an intercept of zero and a slope of 1.27 × 10^11^ carriers per Volt. The error bars represent the standard deviation across eight devices.

Figure 2a,b show current transients, recorded for the SubPc-C_60_ device of Fig. 1b, as a function of illumination intensity. As the illumination is increased (leading to a larger *V*_OC_), the total current extracted from the device increases. Figure 2a shows the current transients for *V*_OC_ values ranging from 3.4 to 37.6 mV while Fig. 2b shows higher *V*_OC_ values from 40.8 to 735 mV (peak *V*_OC_ values for each scan in Fig. 2 are listed in the Supplementary Note 1). Fig. 2c shows the current transients integrated with respect to time yielding the number of charges stored in the device for a given photovoltage. Due to the fast extraction of carriers in the charge extraction experiment, Fig. 2c is the measured number of carriers in the OPV as a function of *V*_OC_ (ref. 31). Thus, with this plot, any measure of photovoltage can be translated into a measure of the number of charge carriers present in the device. While it may be assumed that some charges will undergo recombination before being extracted during the charge extraction measurement, the characteristic extraction time of about 5 μs is significantly shorter than the characteristic recombination time shown in Fig. 1d. Thus, we conclude that the impact of charge carrier recombination on the charge extraction measurement is negligible, in agreement with a previous analysis^31^.

### Extracting the exciton diffusion length

**Figure 3:**
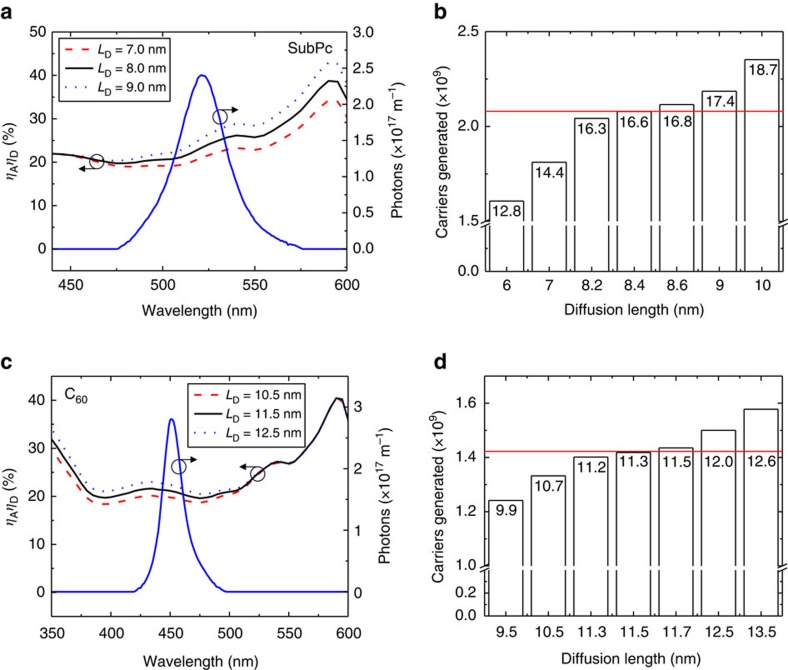
Extracting *L*_D_ from the number of carriers measured by photovoltage. (**a**) Simulated *η*_A_*η*_D_ for variable values of the SubPc *L*_D_, with a constant C_60_ *L*_D_ of 11.5 nm. Also shown, is the total number of photons incident on the device over a 10 μs pulse (solid blue line). (**b**) The number of charge carriers predicted to be generated for different SubPc *L*_D_ values. The horizontal line (red) marks the experimentally measured number of charge carriers generated. The labels on the bars represent the photovoltage (in millivolts) the device would generate for each value of *L*_D,_ showing the sensitivity of the technique. (**c**) Simulated *η*_A_*η*_D_ for variable values of the C_60_ *L*_D_, with a constant SubPc *L*_D_ of 8.4 nm, and the total number of incident photons over a 10 μs pulse (solid blue line). (**d**) The predicted number of generated charge carriers for different values of the C_60_ *L*_D_. The horizontal line (red) marks the experimentally measured number of charge carriers generated, while the bar labels represent the generated photovoltage (in millivolts) for each value of *L*_D_.

With an experimental relationship between the photovoltage and the number of carriers (Fig. 2c), as well as the knowledge that recombination can be ignored, *L*_D_ can be determined for SubPc from the transient photovoltage data of Fig. 1d. The optical pump pulse spectrum is measured with a spectrometer, and the pulse power is measured using a silicon photodiode to determine that the green (*λ*_peak_=530 nm) pulse in Fig. 3a contains 8.64 × 10^9^ photons. Some of the excitons generated from the pulse reach the interface and dissociate into charge carriers, leading to the observed voltage rise in Fig. 1d. The data in Fig. 2c can then be referenced to determine that 2.09 × 10^9^ carriers are generated for the observed voltage plateau of 16.6 mV. Since non-geminate recombination is negligible, this also means that 2.09 × 10^9^ excitons were dissociated into separated charge carriers.

To extract *L*_D_ from the measured number of dissociated excitons, the absorption efficiency (*η*_A_) and the diffusion efficiency (*η*_D_) must also be known. A transfer matrix formalism is used to calculate *η*_A_ and the exciton generation profile as a function of incident photon wavelength (*λ*) (ref. 20). From the generation profile, the exciton diffusion equation can be used to calculate *η*_D_(*λ*, *L*_D_). Example calculations of *η*_A_*η*_D_ are shown in Fig. 3a for varying values of the SubPc *L*_D_. Multiplying *η*_A_*η*_D_ by the number of incident photons (*P*_inc_) and integrating yields the number of carriers that would be generated by exciton dissociation (*n*_gen_) as a function of *L*_D_:


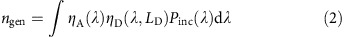


Figure 3b is obtained by applying equation (2) to simulated *η*_A_*η*_D_ traces like the ones shown in Fig. 3a. When the correct value of *L*_D_ is used, the integral of equation (2) matches the measured number of excitons dissociated (2.09 × 10^9^ as determined from the photovoltage transient), and the diffusion length is determined. In this example, the *L*_D_ of SubPc is determined to be 8.4 nm.

The same analysis can be performed for C_60_ using the C_60_ voltage transient of Fig. 1d. The LED pump spectrum is shown in Fig. 3c along with a series of simulated *η*_A_*η*_D_ curves. When equation (2) is applied to the curves in Fig. 3c, Fig. 3d is generated. In this example, the diffusion length of C_60_ is found to be 11.5 nm for the *V*_OC_ transient of Fig. 1d.

Figure 3b,d demonstrate the sensitivity of the photovoltage-based approach. Each bar is labelled by the photovoltage (in mV) that would be measured for the corresponding *L*_D_ and number of charge carriers generated. The oscilloscope employed here to measure voltage has a resolution of ∼0.2 mV which gives an *L*_D_ resolution of about±0.2 nm for the device in Fig. 1b. While the diffusion length can be determined within this small error for a given device, there remain uncontrolled effects in device fabrication that lead to more substantial device-to-device deviations. As such, the standard deviation is taken as the error in *L*_D_. By averaging across twelve devices, *L*_*D*_ values of (8.5±1.0) nm and (11.5±1.2) nm are obtained for SubPc and C_60_, respectively. Active layer thicknesses for all devices considered in this study are included in the Supplementary Note 2.

**Figure 4:**
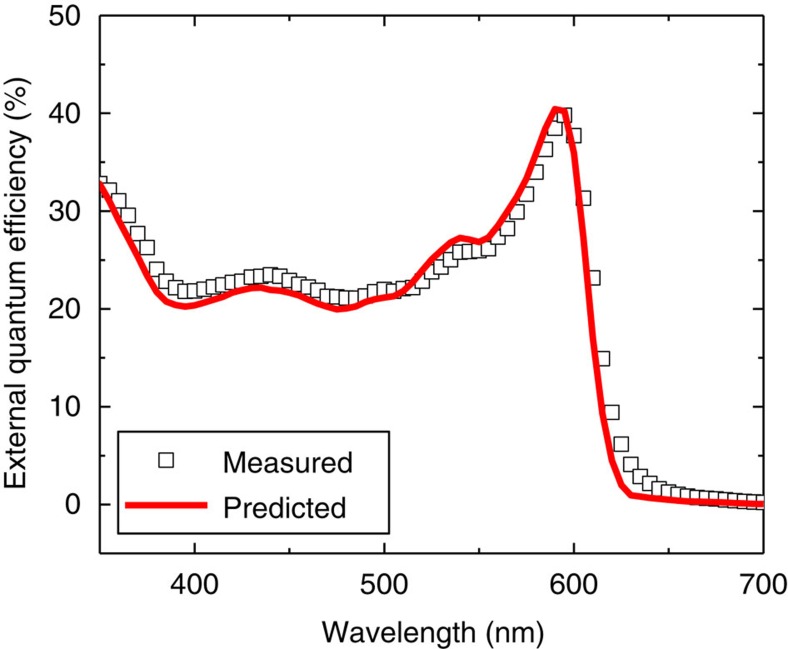
Comparing photovoltage-determined values of *L*_D_ to *η*_EQE_. Comparison of the measured device *η*_EQE_ to that predicted using *L*_D_ values extracted from photovoltage with *η*_CC_=1. Since the measurement of *L*_D_ intrinsically reflects the impact of geminate recombination, the agreement between the measured and simulated *η*_EQE_ suggests that non-geminate recombination is negligible in this device at short circuit.

To put these values into context, the *η*_EQE_ was also measured for the SubPc-C_60_ device of Fig. 1b and is shown in Fig. 4. When this *η*_EQE_ data is compared with a prediction using an optical transfer matrix formalism and exciton diffusion equation with the photovoltage determined values of *L*_*D*_^20^, excellent agreement is observed. Direct fitting of the *η*_EQE_ data using the same model yields *L*_D_ values of 8.0 and 12.2 nm for SubPc and C_60_, respectively, similar to those obtained from photovoltage. This agreement occurs despite the fact that the measurement of *η*_EQE_ may include non-geminate recombination losses, which are circumvented in the photovoltage measurement. These results suggest that non-geminate recombination losses are negligible in planar heterojunction OPVs based on SubPc-C_60_ near short-circuit. Thus, the charge collection efficiency at short-circuit is dominated by geminate recombination losses^34^.

It is instructive to compare the photovoltage-extracted *L*_*D*_ for SubPc to that obtained from conventional thickness-dependent PL quenching. PL quenching involves the tracking of PL as a measure of excitons that do not reach the D–A interface. This measurement is subtly different than the photovoltage approach in that the photovoltage is only sensitive to excitons that reach the interface, dissociate, and yield charge carriers that contribute to the voltage. Indeed, while excitons that form charge transfer states and undergo rapid geminate recombination will not contribute substantially to the photovoltage, these states will contribute to a reduction in PL. As such, materials and architectures that exhibit rapid geminate recombination should yield a different value of *L*_D_ depending on the method applied. Previous reports for SubPc place its diffusion length in the range of *L*_D_=10.7 nm, larger than the results reported here using photovoltage^3^. As noted, this disagreement suggests that there is substantial geminate recombination present at the D–A interface, and that the value extracted from photovoltage is as a result, an underestimate to the intrinsic *L*_*D*_ of SubPc. It is worth noting that in materials with substantial exciton-exciton or exciton-polaron quenching, these losses will also be reflected in *L*_*D*_. Here no variation in *L*_D_ is observed with active layer thickness or light intensity^35^. In cases where geminate recombination plagues a planar heterojunction device of interest, the extracted *L*_D_ still represents the extractable fraction of excitons dissociating at the D–A heterojunction.

### Measuring dark exciton transport in phthalocyanine devices

While the discussion hereto has focused on the luminescent donor material SubPc to demonstrate the photovoltage technique, the method is broadly applicable provided the material of interest can be used as either a donor or acceptor layer in a planar heterojunction OPV (*id est*, it produces a photovoltage) without need to optimize the device or reach high efficiency.

**Figure 5:**
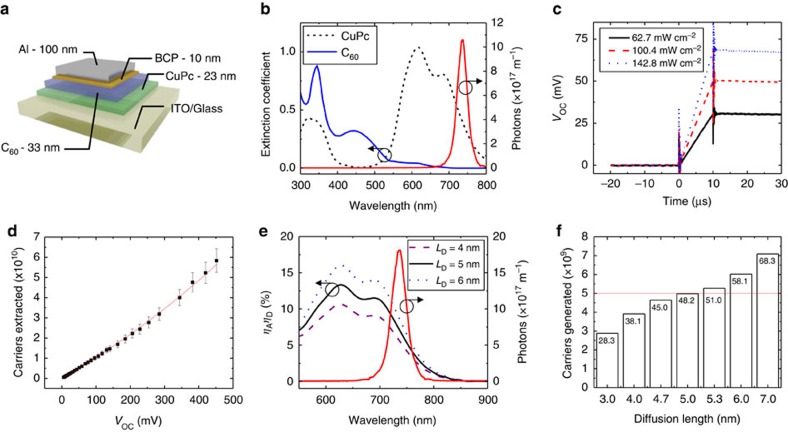
Measuring the *L*_D_ of CuPc. (**a**) Architecture for the planar heterojunction OPV based on the donor-acceptor pairing of CuPc-C_60_. (**b**) Comparison of the extinction coefficients for CuPc and C_60_ as well as the spectrum of the LED pulse (*λ*_peak_=735 nm) used to pump CuPc. (**c**) Photovoltage rises recorded when pumping CuPc with the *λ*=735 nm LED at intensities of 62.7 mW cm^−2^ (black), 100.4 mW cm^−2^ (red) and 142.8 mW cm^−2^ (blue). (**d**) Relationship between charge carriers and voltage in the CuPc-C_60_ device obtained using the charge extraction method and an exponential fit to the data. The error bars represent the standard deviation across eight devices. (**e**) Simulated *η*_A_*η*_D_ curves for three CuPc *L*_D_ values with a constant C_60_ *L*_D_ of 12.1 nm compared with the time integrated LED pump spectrum. (**f**) Comparison of the predicted number of charge carriers generated (for multiple values of the CuPc *L*_D_) to the photovoltage-based measurement (horizontal line). The *V*_OC_ (in millivolts) that would be measured for the corresponding number of charge carriers is labelled for each bar.

As demonstrated with C_60_, the photovoltage technique is capable of probing the *L*_D_ of dark excitons. Here, we further demonstrate this by extracting the *L*_D_ of the archetypal donor material copper phthalocyanine (CuPc). Figure 5a shows the device structure used, consisting of a heterojunction between CuPc and C_60_, and Fig. 5b demonstrates that nearly all of the pump LED (*λ*_peak_=735 nm) is absorbed by CuPc. Three voltage transients for 10 μs pulses of varying intensity are shown in Fig. 5c. As the intensity of the LED pulse increases, the photovoltage response of the device plateaus at larger voltages. Using Fig. 5d this can be related to an increased number of charge carriers being generated at the D–A interface. As demonstrated for SubPc and C_60_, the *L*_D_ can be extracted using Fig. 5c,d. Figure 5e shows the calculated *η*_A_*η*_D_ for three possible *L*_D_ values overlaid with the LED spectrum at an intensity of 100.4 mW cm^−2^. Figure 5f gives the results of applying equation (2) to a series of these *η*_A_*η*_D_ curves for the pump shown in Fig. 5e. In this example, a diffusion length of 5.0 nm is extracted for CuPc. This value agrees well with the *L*_D_ extracted for the other pump intensities shown in Fig. 5c: *L*_D_=4.94 nm for 62.7 mW cm^−2^ and *L*_D_=4.97 nm for 142.8 mW cm^−2^.

**Figure 6:**
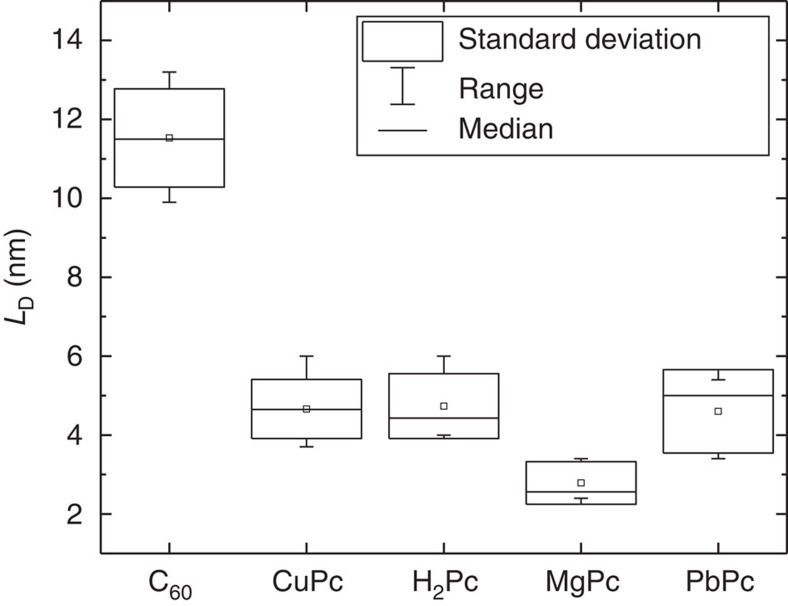
Comparison of *L*_D_ values obtained using photovoltage. Values of *L*_D_ extracted using the photovoltage-based technique for C_60_ and a series of phthalocyanine molecules differing in the coordinating metal atom: metal-free (H_2_Pc), magnesium (MgPc), copper (CuPc) and lead (PbPc). The small square symbol represents the mean of all measured devices and the boxes represent the standard deviation across all measured devices.

Figure 6 shows the *L*_D_ extracted for a variety of dark materials using photovoltage. In addition to C_60_, a series of phthalocyanine molecules differing only in the central coordinating metal atom are shown: metal-free (H_2_Pc) *L*_D_=4.7 nm (Supplementary Fig. 1), magnesium (MgPc) *L*_D_=2.8 nm (Supplementary Fig. 2), copper (CuPc) *L*_D_=4.7 nm, and lead (PbPc) *L*_D_=4.6 nm (Supplementary Fig. 3). Besides demonstrating the wide applicability of the photovoltage-based *L*_D_ technique, Fig. 6 demonstrates an interesting trend. The average *L*_D_ of all of the phthalocyanine molecules fall within a range of about two nanometers despite the nature of the exciton in these molecules differing as a function of the coordinating metal atom. The exciton in H_2_Pc is likely a singlet exciton with a lifetime of about 250 ps, whereas the excitons in CuPc and PbPc are likely long-lived triplet excitons^24^. The sharing of a crystal structure between H_2_Pc and CuPc further makes the similar exciton diffusion length of interest as these two materials provide a unique opportunity to contrast the motion of singlet and triplet excitons in very similar environments.

This work demonstrates an alternate, photovoltage-based method for probing exciton harvesting that is applicable to any material that can facilitate exciton dissociation at the heterojunction in a planar OPV. The approach utilizes a unique understanding of the information contained in the photovoltage of an OPV to measure the number of charge carriers in a device before any substantial non-geminate recombination has occurred. For devices that are still subject to geminate recombination, the measured values of *L*_D_ represent the fraction of excitons which are able to be dissociated and extracted as photocurrent. Photovoltage thus provides a useful tool to probe exciton harvesting in dark excitonic materials which are inaccessible by conventional PL quenching techniques, while also offering insight into the recombination mechanisms responsible for non-unity charge collection efficiency.

## Methods

### Device preparation

Devices were fabricated on glass substrates, pre-patterned with a 150-nm-thick layer of indium-tin-oxide with a sheet resistance of 15 Ω per square. Each substrate was degreased by sonication in tergitol and acetone followed by boiling in isopropyl alcohol. Before thin film deposition, substrates were exposed to an ultraviolet-ozone atmosphere for 10 min. All device layers were fabricated using vacuum thermal sublimation/evaporation at pressures below 10^−6^ Torr. The organic layers were deposited at 0.2 nm s^−1^, and the Al cathode was deposited at 0.3 nm s^−1^. All materials were used as received. Materials were obtained from the following suppliers: H_2_Pc and MgPc from Sigma-Aldrich; CuPc from Acros Organics; lead phthalocyanine from TCI America; BCP and Al from Alfa Aesar; SubPc from Luminescence Technology Corporation; and C_60_ from MER Corporation. All devices were capped with a 10-nm-thick exciton blocking layer of BCP and a 100-nm-thick cathode layer of Al. All devices have an active area of 0.25 cm^2^.

### Charge extraction

Charge extraction experiments were performed in atmosphere using an M455F1, M530F1, M625F1 or M735L3 LED from Thorlabs as the light source. The n-channel MOSFETs used to switch the LED and the OPV were manufactured by STmicroelectronics (STP27N3LH5). Integrated gate driver circuits were used to simultaneously switch the LED transistor and the OPV transistor. Since the transistors controlling the LED and the OPV must switch states in opposite directions at the same time, an inverting gate driver (Microchip Technology TC4421AVPA) was connected to the LED transistor and a non-inverting gate driver (Microchip Technology TC4422AVPA) was connected to the OPV transistor. The gate drivers were operated by the same pulses from an Agilent 33220A pulse generator, and the current transients were recorded using a Tektronix TDS5104B oscilloscope.

### Voltage transients

Voltage transients were measured in atmosphere. The same LEDs used in the charge extraction experiment were used to obtain the voltage transients. The LEDs were powered by a Hewlett-Packard 8114A pulse generator, and the voltage transients were recorded by a Tektronix TDS5104B oscilloscope. The spectrum of each LED was measured using an Ocean Optics HR4000 spectrometer and the power output of the LEDs was recorded using a Thorlabs PDA36A amplified silicon photodetector. The values of *L*_D_ obtained were (11.5±1.2) nm for C_60_, (8.5±1.0) nm for SubPc, (4.7±0.7) nm for H_2_Pc, (2.8±0.4) nm for MgPc, (4.7±0.7) nm for CuPc, and (4.6±0.9) nm for PbPc. Values of *L*_D_ for a single device were determined by averaging three to five voltage transient measurements using LED intensities ranging from about 10 to 150 mW cm^−2^. Reported *L*_D_ values are obtained by averaging measurements of twelve C_60_, twelve SubPc, six H_2_Pc, three MgPc, fourteen CuPc and three PbPc devices. The error is the standard deviation across all devices and LED intensities.

### Computational notes

The electric field in the device (used to extract *L*_D_ from photovoltage measurements and to fit *η*_EQE_) was calculated using a transfer matrix formalism previously described^20^. The layer thicknesses and optical constants needed as inputs to the model were measured using spectroscopic ellipsometry. The boundary conditions used to solve the exciton diffusion equation were a reflecting indium-tin-oxide–donor interface and an exciton sink at the donor-acceptor interface consistent with PL quenching-based measurements of *L*_D_.

### Data availability

Data from this work is available from the corresponding author upon request.

## Additional information

**How to cite this article:** Mullenbach, T. K. *et al*. Probing dark exciton diffusion using photovoltage. *Nat. Commun.* **8,** 14215 doi: 10.1038/ncomms14215 (2017).

**Publisher's note:** Springer Nature remains neutral with regard to jurisdictional claims in published maps and institutional affiliations.

## Supplementary Material

Supplementary Information Supplementary Figures 1–3 and Supplementary Notes 1–2

## References

[b1] Tang C. W. Two-layer organic photovoltaic cell. Appl. Phys. Lett. 48, 183–185 (1986).

[b2] Peumans P., Yakimov A. & Forrest S. R. Small molecular weight organic thin-film photodetectors and solar cells. J. Appl. Phys. 93, 3693–3723 (2003).

[b3] Menke S. M., Luhman W. A. & Holmes R. J. Tailored exciton diffusion in organic photovoltaic cells for enhanced power conversion efficiency. Nat. Mater. 12, 152–157 (2013). 23142837 10.1038/nmat3467

[b4] Zhang M., Wang H., Tian H., Geng Y. & Tang C. W. Bulk heterojunction photovoltaic cells with low donor concentration. Adv. Mater. 23, 4960–4964 (2011). 21956229 10.1002/adma.201102173

[b5] Gregg B. A. Excitonic solar cells. J. Phys. Chem. B 107, 4688–4698 (2003).

[b6] Schlenker C. W. . Cascade organic solar cells. Chem. Mater. 23, 4132–4140 (2011).

[b7] Mikhnenko O. V., Blom P. W. M. & Nguyen T. Q. Exciton diffusion in organic semiconductors. Energy Environ. Sci. 8, 1867–1888 (2015).

[b8] Cnops K. . 8.4% efficient fullerene-free organic solar cells exploiting long-range exciton energy transfer. Nat. Commun. 5, 3406 (2014). 24603622 10.1038/ncomms4406

[b9] Yu G., Gao J., Hummelen J. C., Wudl F. & Heeger A. J. Polymer photovoltaic cells: enhanced efficiencies via a network of internal donor- acceptor heterojunctions. Science 270, 1789–1791 (1995).

[b10] Menke S. M. & Holmes R. J. Exciton diffusion in organic photovoltaic cells. Energy Environ. Sci. 7, 499–512 (2014).

[b11] Luhman W. A. & Holmes R. J. Investigation of energy transfer in organic photovoltaic cells and impact on exciton diffusion length measurements. Adv. Funct. Mater. 21, 764–771 (2011).

[b12] Lunt R. R., Giebink N. C., Belak A. A., Benziger J. B. & Forrest S. R. Exciton diffusion lengths of organic semiconductor thin films measured by spectrally resolved photoluminescence quenching. J. Appl. Phys. 105, 053711 (2009).

[b13] Mullenbach T. K., McGarry K. A., Luhman W. A., Douglas C. J. & Holmes R. J. Connecting molecular structure and exciton diffusion length in rubrene derivatives. Adv. Mater. 25, 3689–3693 (2013). 23754475 10.1002/adma.201300641

[b14] Gregg B. A., Sprague J. & Peterson M. W. Long-range singlet energy transfer in perylene bis(phenethylimide) films. J. Phys. Chem. B 101, 5362–5369 (1997).

[b15] Theander M. . Photoluminescence quenching at a polythiophene/C_60_ heterojunction. Phys. Rev. B 61, 12957–12963 (2000).

[b16] Shaw P. E., Ruseckas A. & Samuel I. D. W. Exciton diffusion measurements in poly(3-hexylthiophene). Adv. Mater. 20, 3516–3520 (2008).

[b17] Scully S. R. & McGehee M. D. Effects of optical interference and energy transfer on exciton diffusion length measurements in organic semiconductors. J. Appl. Phys. 100, 034907 (2006).

[b18] Kroeze J. E. . Contactless determination of the photoconductivity action spectrum, exciton diffusion length, and charge separation efficiency in polythiophene-sensitized TiO2 bilayers. J. Phys. Chem. B 107, 7696–7705 (2003).

[b19] Kozlov O. V. . Real-time tracking of singlet exciton diffusion in organic semiconductors. Phys. Rev. Lett. 116, 1–5 (2016). 10.1103/PhysRevLett.116.057402 26894732

[b20] Pettersson L. A. A., Roman L. S. & Inganas O. Modeling photocurrent action spectra of photovoltaic devices based on organic thin films. J. Appl. Phys. 86, 487–496 (1999).

[b21] Lunt R. R., Benziger J. B. & Forrest S. R. Relationship between crystalline order and exciton diffusion length in molecular organic semiconductors. Adv. Mater. 22, 1233–1236 (2010). 20437510 10.1002/adma.200902827

[b22] Menke S. M., Mullenbach T. K. & Holmes R. J. Directing energy transport in organic photovoltaic cells using interfacial exciton gates. ACS Nano 9, 4543–4552 (2015). 25798712 10.1021/acsnano.5b01160

[b23] Markov D. E., Amsterdam E., Blom P. W. M., Sieval A. B. & Hummelen J. C. Accurate measurement of the exciton diffusion length in a conjugated polymer using a heterostructure with a side-chain cross-linked fullerene layer. J. Phys. Chem. A 109, 5266–5274 (2005). 16839049 10.1021/jp0509663

[b24] Caplins B. W., Mullenbach T. K., Holmes R. J. & Blank D. A. Intermolecular interactions determine exciton lifetimes in neat films and solid state solutions of metal-free phthalocyanine. J. Phys. Chem. C. 119, 27340–27347 (2015).

[b25] Luhman W. A. & Holmes R. J. Enhanced exciton diffusion in an organic photovoltaic cell by energy transfer using a phosphorescent sensitizer. Appl. Phys. Lett. 94, 153304 (2009).

[b26] Pandey R. & Holmes R. J. Characterizing the charge collection efficiency in bulk heterojunction organic photovoltaic cells. Appl. Phys. Lett. 100, 15–18 (2012).

[b27] Giebink N. C., Wiederrecht G. P., Wasielewski M. R. & Forrest S. R. Ideal diode equation for organic heterojunctions. I. Derivation and application. Phys. Rev. B 82, 155305 (2010).

[b28] Cheyns D. . Analytical model for the open-circuit voltage and its associated resistance in organic planar heterojunction solar cells. Phys. Rev. B 77, 165332 (2008).

[b29] Mullenbach T. K. & Holmes R. J. Relating photocurrent, photovoltage, and charge carrier density to the recombination rate in organic photovoltaic cells. Appl. Phys. Lett. 107, 123303 (2015).

[b30] Pivrikas A., Sariciftci N. S., Juška G. & Österbacka R. A review of charge transport and recombination in polymer/fullerene organic solar cells. Prog. Photovoltaics Res. Appl. 15, 677–696 (2007).

[b31] Shuttle C. G. . Charge extraction analysis of charge carrier densities in a polythiophene/fullerene solar cell: Analysis of the origin of the device dark current. Appl. Phys. Lett. 93, 183501 (2008).

[b32] Peter L. M., Duffy N. W., Wang R. L. & Wijayantha K. G. U. Transport and interfacial transfer of electrons in dye-sensitized nanocrystalline solar cells. J. Electroanal. Chem. 524–525, 127–136 (2002).

[b33] Duffy N. W., Peter L. M., Rajapakse R. M. G. & Wijayantha K. G. U. A novel charge extraction method for the study of electron transport and interfacial transfer in dye sensitised nanocrystalline solar cells. Electrochem. Commun. 2, 658–662 (2000).

[b34] Credgington D., Jamieson F. C., Walker B., Nguyen T. Q. & Durrant J. R. Quantification of geminate and non-geminate recombination losses within a solution-processed small-molecule bulk heterojunction solar cell. Adv. Mater. 24, 2135–2141 (2012). 22431341 10.1002/adma.201104738

[b35] Verreet B. . Reducing exciton-polaron annihilation in organic planar heterojunction solar cells. Phys. Rev. B 90, 115304 (2014).

