# Supplementary material for: Probing dark exciton diffusion using photovoltage
Source: Nat Commun. 2017 Jan 27;8:14215. doi: 10.1038/ncomms14215 (PMC5290169; doi:10.1038/ncomms14215)
Supplement: Supplementary Information — Supplementary Figures 1–3 and Supplementary Notes 1–2 [file ncomms14215-s1.pdf]

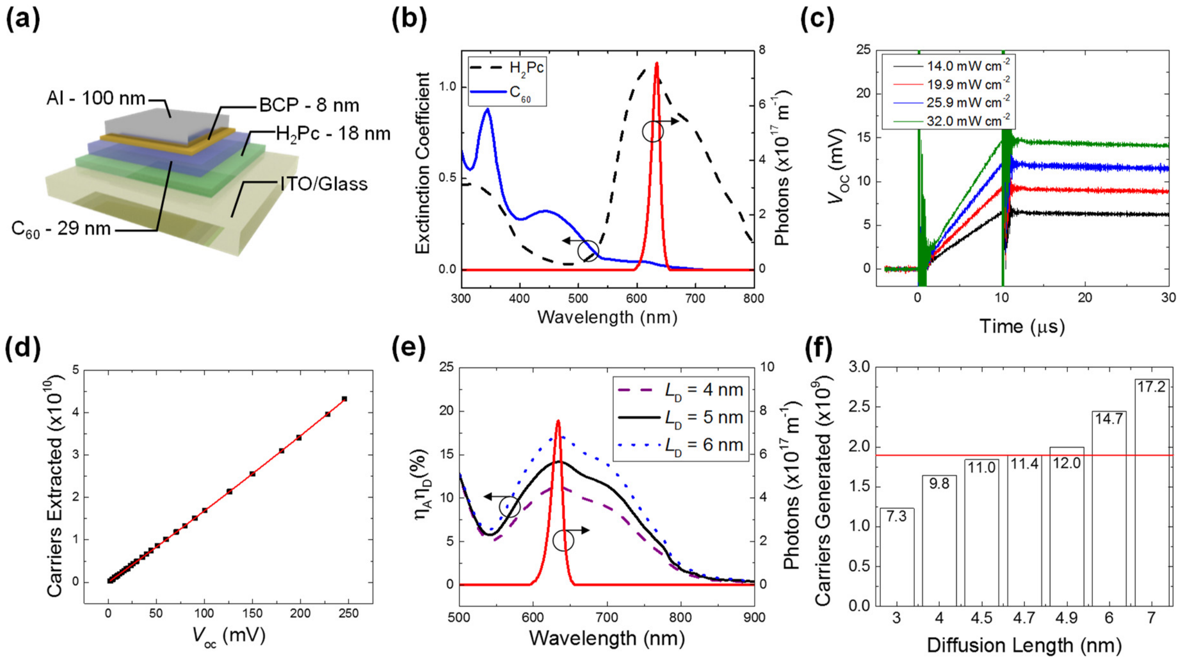

Supplementary Figure 1 Measuring the L_D of H_2Pc: (a) Architecture for the planar heterojunction organic photovoltaic cell (OPV) based on the donor-acceptor pairing of metal-free phthalocyanine (H_2Pc)-C_{60}. (b) Comparison of the extinction coefficients for H_2Pc and C_{60} as well as the spectrum of the LED pulse (λ_{peak} = 625 nm) used to pump H_2Pc. (c) Four photovoltage rises recorded when pumping H_2Pc with the λ = 625 nm LED at intensities of 14.0 mW cm^{-2} (black), 19.9 mW cm^{-2} (red), 25.9 mW cm^{-2} (blue) and 32.0 mW cm^{-2} (green). (d) The relationship between charge carriers and voltage for the H_2Pc-C_{60} device obtained using the charge extraction method and a linear fit to the data. (e) Simulated $\eta_A\eta_D$ curves for three H_2Pc L_D values compared to the time integrated LED pump spectrum. (f) Comparison of the predicted number of charge carriers generated (for multiple values of the H_2Pc L_D) to the photovoltage-based measurement (horizontal line). The V_{OC} (in millivolts) that would be measured for the corresponding number of charge carriers is labeled for each bar.

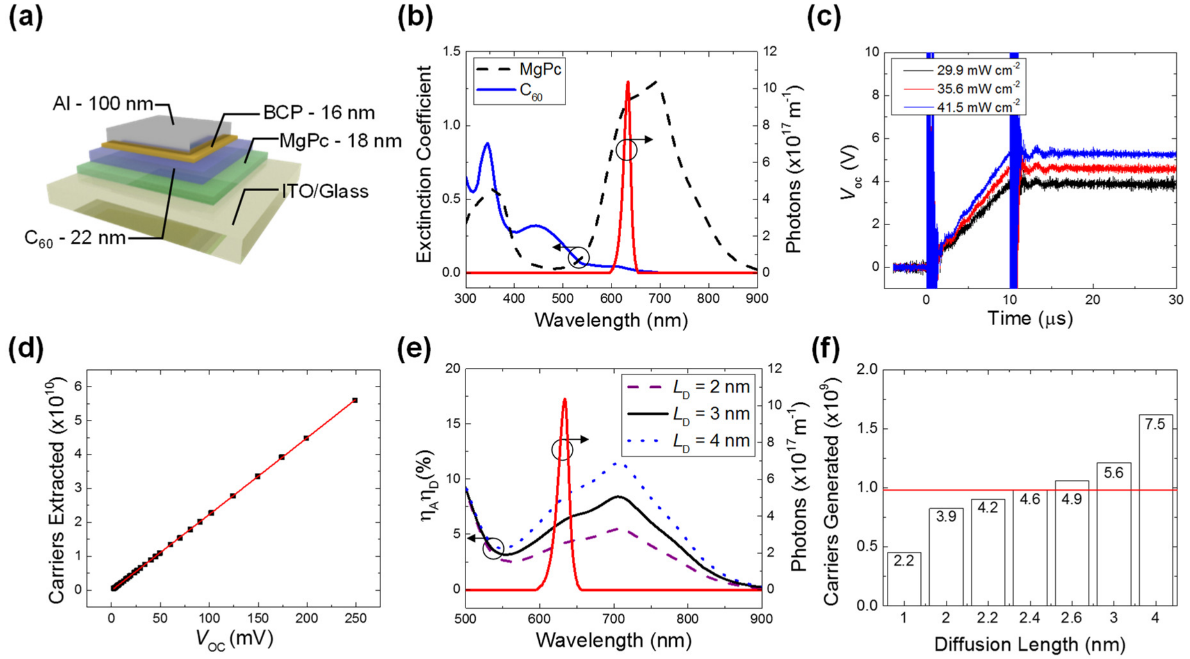

Supplementary Figure 2 Measuring the L_D of MgPc: (a) Architecture for the planar heterojunction OPV based on the donor-acceptor pairing of magnesium phthalocyanine (MgPc)-C_{60}. (b) Comparison of the extinction coefficients for MgPc and C_{60} as well as the spectrum of the LED pulse (λ_{peak} = 625 nm) used to pump MgPc. (c) Three photovoltage rises recorded when pumping MgPc with the λ = 625 nm LED at intensities of 29.9 mW cm^{-2} (black), 35.6 mW cm^{-2} (red), 41.5 mW cm^{-2} (blue). (d) The relationship between charge carriers and voltage for the MgPc-C_{60} device obtained using the charge extraction method and a linear fit to the data. (e) Simulated $\eta_A\eta_D$ curves for three MgPc L_D values compared to the time integrated LED pump spectrum. (f) Comparison of the predicted number of charge carriers generated (for multiple values of the MgPc L_D) to the photovoltage-based measurement (horizontal line). The V_{OC} (in millivolts) that would be measured for the corresponding number of charge carriers is labeled for each bar.

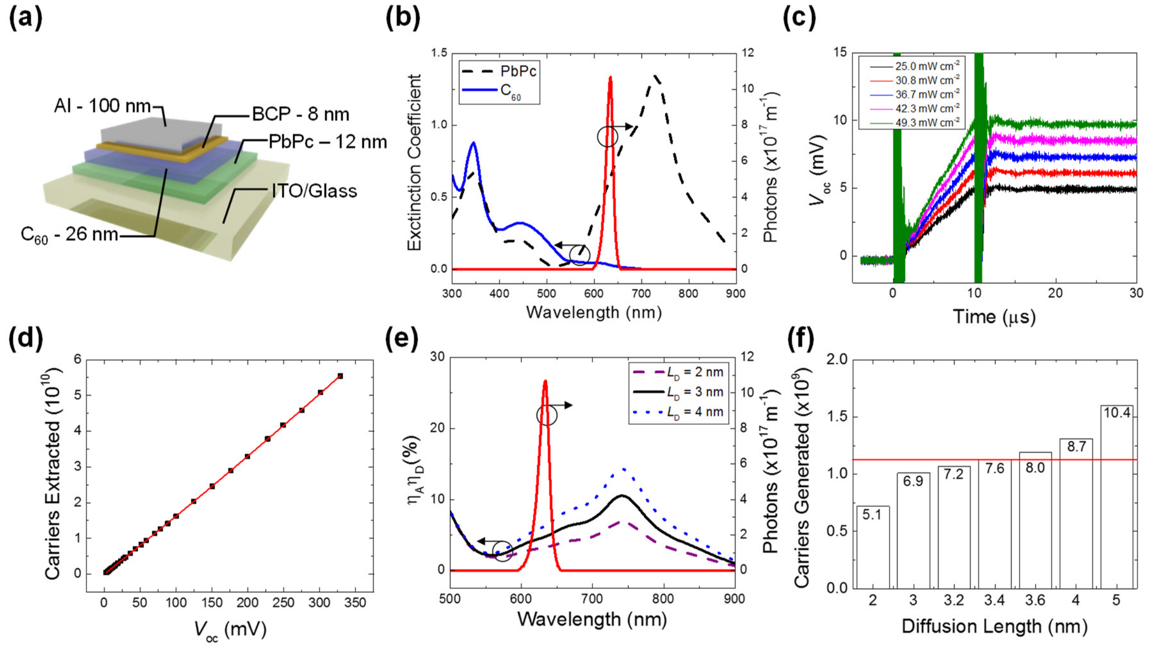

Supplementary Figure 3 Measuring the L_D of PbPc: (a) Architecture for the planar heterojunction OPV based on the donor-acceptor pairing of lead phthalocyanine (PbPc) -C_{60}. (b) Comparison of the extinction coefficients for PbPc and C_{60} as well as the spectrum of the LED pulse (λ_{peak} = 625 nm) used to pump PbPc. (c) Five photovoltage rises recorded when pumping PbPc with the λ = 625 nm LED at intensities of 25.0 mW cm^{-2} (black), 30.8 mW cm^{-2} (red), 36.7 mW cm^{-2} (blue), 42.3 mW cm^{-2} (pink) and 49.3mW cm^{-2} (green). (d) The relationship between charge carriers and voltage in the PbPc-C_{60} device obtained using the charge extraction method and an exponential fit to the data. (e) Simulated $\eta_A\eta_D$ curves for three PbPc L_D values compared to the time integrated LED pump spectrum. (f) Comparison of the predicted number of charge carriers generated (for multiple values of the PbPc L_D) to the photovoltage-based measurement (horizontal line). The V_{OC} (in millivolts) that would be measured for the corresponding number of charge carriers is labeled for each bar.

Supplementary Note 1 - Additional data for current transients shown in Figure 2a and Figure 2b:

In Fig. 2a and Fig. 2b of the text, each pictured current transient has a corresponding steady-state V_{OC} that is established across the device prior to charge extraction. These measured V_{OC} values are:

Figure 2a (mV): 3.4, 4, 5.4, 6.3, 7.1, 8.3, 9.5, 11.0, 12.8, 14.6, 16.2, 18.6, 19.6, 22.2, 25.6, 29.2, 37.6

Figure 2b (mV): 40.8, 45.2, 50.2, 63.0, 70.0, 80.0, 89.2, 100.6, 125.6, 147.5, 172.5, 201.5, 247.0, 307.5, 355.0, 410.0, 504.0, 601.0, 701.0 735.0

Supplementary Note 2 - Device thicknesses:

The donor/acceptor layer thicknesses for all devices considered in this study are:

- SubPc/C_{60} (nm): 15/11, 14/10, 19/8, 15/19, 18/16, 14/33, 12/29, 16/27, 21/27, 47/29, 21/30, 24/36
- CuPc/C_{60} (nm): 16/44, 16/45, 23/21, 14/12, 18/21, 19/13, 11/33, 12/26, 20/50, 20/40, 21/25, 13/28, 17/29, 15/39
- H_2Pc/C_{60} (nm): 27/21, 18/14, 23/28, 18/29, 23/24, 15/25
- MgPc/C_{60} (nm): 11/28, 24/22, 18/22
- PbPc/C_{60} (nm): 19/36, 20/40, 12/26
